# Notes

**Published:** 1898-08-27

**Authors:** 


### NOTES.

The new Jenny Lind Infirmary for Sick Children is now actually in hand. The scheme was propounded by Norwich and Norfolk as a means of celebrating the Diamond Jubilee. The committee have been able to accept a contract for the whole of the new infirmary, with the exception of one wing, and at the same time to observe the condition laid down by the Earl of Leicester—that nothing shall be built which it is not possible also to endow. There is a good deal of money still wanted; but a start has been made, and there is now every prospect of the little patients being in their new home before December, 1899. The old house in Pottergate Street was never intended for a hospital, and it fell under the condemnation of the medical staff. By way of honouring the memory of the late Mrs. Colman, Mr. J. J. Colman came forward with the gift of a new site on Unthank's Road; and at a meeting of city and county people, held at the Guildhall, it was resolved that the erection and endowment of a new infirmary thereon would be a fitting enterprise with which to celebrate the Diamond Jubilee. Lord Leicester led off with a contribution of £2,000, followed up with another thousand for endowment purposes only. This fund now stands at about £9,500. The sale of "brick books," it is hoped, will realise some £500. The sale of the old site in Pottergate Street produced £2,000. A bequest of £1,500 has been received under the will of the late Mr. Richard England, while £1,000 was bequeathed by Mr. Ernest England in order to endow a cot. The chairman of the Jenny Lind Committee holds a promise for £250. There is a Norfolk lady who gives £50 on condition that nine other Norfolk ladies will follow suit. At present seven fifties have come to hand, leaving three fifties still to be raised in order to fulfil the conditions of the gift. The estimate of the committee is, roughly, that £12,000 is wanted for building and furnishing purposes, and another £10,000 for endowment; and as the money promised or in hand amounts in all to £16,500, there is still £5 000 or £6,000 to be obtained from some source or other before the building assumes its final form. The work at present in hand comprises only the administration block and one wing, the other wing necessary to complete the building depending on the good pleasure of philanthropic people to whom the work of the Jenny Lind Infirmary commends itself.

